# Biosolubility of high temperature insulation wools in simulated lung fluids

**DOI:** 10.1186/s12995-019-0235-z

**Published:** 2019-05-14

**Authors:** Annapaola Cannizzaro, Federica Angelosanto, Elena Barrese, Antonella Campopiano

**Affiliations:** 1 Department of Medicine, Epidemiology, Occupational and Environmental Hygiene, National Institute for Insurance against Accidents at Work-INAIL Research Area, Via Fontana Candida, 00078 Monte Porzio Catone, Rome Italy; 2 Department of Medicine, Epidemiology, Occupational and Environmental Hygiene, National Institute for Insurance against Accidents at Work, 88046 Lamezia Terme, Catanzaro, Italy

**Keywords:** Alkaline earth silicate wool, Biosolubility, Man-made mineral fibers, Polycrystalline wools, Scanning electron microscopy

## Abstract

**Objective:**

Biosolubility is an important parameter in the understanding of mechanisms involved in pulmonary toxicity of fibrous materials. It can be studied in vitro using models of simulated lung fluids and observing the loss of structural molecules, expressed as dissolution constant (K_dis_). The aim of this paper was the study of dissolution behaviour of four wools belonging to high temperature insulation wools (HTIW) in saline solutions simulating lung fluids.

**Methods:**

Four HTIW were studied in saline solutions at pH 7.4 (representative of the extracellular environment) and 4.5 (representative of the intracellular conditions): refractory ceramic fibers (RCF), two alkaline earth silicate wools (AES1 and AES2 with high calcium and magnesium content respectively), and polycrystalline wools (PCW). Size, morphological and chemical changes of fibers were observed by scanning electron microscopy (SEM) with energy-dispersive X-ray spectrometry (EDS) and inductively coupled plasma atomic emission spectrometry (ICP-AES).

**Results:**

RCF, AES2 and PCW did not show statistically significant diameter changes. AES1 size distribution shifted to a larger mean diameter suggesting that through dissolution there was a preferential loss of thin fibers at acid pH after 14 days of treatment.

Both AES wools showed selective leaching of alkali/alkali earth oxides (incongruent dissolution) at pH 7.4: a fast and extensive selective leaching of calcium for AES1 with complete dissolution of fibers already after 14 days of treatment and a moderate selective leaching of magnesium for AES2. PCW showed some transversal breakage of the fibers in both pH environments (low congruent dissolution). For RCF, the treatment produced uncorroded fibers in both pH environments without chemical changes and fiber fragmentation (no dissolution).

The estimated K_dis_ at physiological pH followed the sequence: AES1 > AES2 > PCW > RCF. All wools had a low K_dis_ at acid pH suggesting a low dissolution rate of short fibers.

**Conclusion:**

The leaching process and transverse fragmentation play an important role in the biopersistence mechanisms and pathogenicity of fibers and the K_dis_ estimate is undoubtedly useful as a preliminary toxicological screening of fibers, especially for developing fibers.

## Background

Asbestos was used widely in Italy until its use was banned in 1992 leading to the development of other fibrous materials [1–3]. Man-made mineral fibers (MMMF) is a wider group of inorganic fibers used widely in commercial and residential buildings for both thermal and sound insulation purposes. High temperature insulation wools (HTIW) are MMMF, used in high temperature industrial applications (600–1800 °C) include: amorphous wools such as refractory ceramic fibers (RCF) and alkaline earth silicate wools (AES) and crystalline wools such as polycrystalline wools (PCW) [4].

RCF fibers have been classified by the International Agency for Research on cancer (IARC) as possibly carcinogenic to humans (Group 2B) [5]. AES wools are materials that are specifically designed to have low biopersistence and therefore low hazardousness [3, 6]. Fibers can be designed to be inherently less hazardous. The development of alkaline earth silicate (AES) wools, with alkaline oxides and alkali earth oxide content less or equal to 18% by weight, determined the birth of a new class of fibers a low biopersistence. The chemical composition of AES wools have been adjusted to minimize possible harm to human health. AES wools are materials that have been designed to be rapidly cleared from lung tissue [2].

IARC decided not to make an overall evaluation because no human data were available, although such fibers appear to have low carcinogenic potential in experimental animals [5].

PCW is an alumina-silicate wool with crystalline structure, used for application temperature greater than 1400 °C and in chemically aggressive environments [4]. In 1988, the IARC included PCW fibers into a broad group termed “ceramic fibers” classified as possible human carcinogens (Group 2B) [7].

According to European Regulations [8–10], the term “ceramic wools” is applied to fibers with alkaline oxides and alkali earth oxide (Na_2_O + K_2_O + CaO + MgO + BaO) content less or equal to 18% by weight, while “mineral wools” is applied to fibers with content of such oxide greater than 18%. The classification as carcinogenic need not apply if the fibers fulfill one of following two notes: “Note R: The classification as a carcinogen need not apply to fibers with a length weighted geometric mean diameter less two standard errors greater than 6 μm” or “Note Q: The classification as a carcinogen need not apply if it can be shown that the substance fulfils one of the following conditions: a short-term biopersistence test by inhalation has shown that the fibers longer than 20 μm have a weighted half life less than 10 days, or a short-term biopersistence test by intratracheal instillation has shown that the fibers longer than 20 μm have a weighted half life less than 40 days, or an appropriate intra-peritoneal test has shown no evidence of excess carcinogenicity, or absence of relevant pathogenicity or neoplastic changes in a suitable long term inhalation test”.

AES wools are exonerated from classification under Note Q.

The potential health hazard of inhalation of fibers is frequently summarized by the shorthand expression “3D” for dose-dimension-durability. 3D paradigm is widely cited in the literature [11–13]. The dose is related to the quantity of inhaled fibers. Fiber dimensions are relevant for two reasons: fiber diameter influences the fiber respirability and fiber length affects the fiber clearance [14–18].

Fibers with diameter < 3 μm, length > 5 μm, and aspect ratio (length to width) greater than or equal to 3, are classified as “respirable fibers” [19]. They reach the target areas of the lung, particularly the alveolar region. Fibers with lengths greater than 20 μm are not engulfed by macrophages, and are more likely to lead to lung injury than shorter fibers that are more readily removed by macrophages. Long and biopersistent fibers deposited in the lung environment can lead to pulmonary diseases [12, 20]. However, a role for short fibers in disease cannot completely be excluded. Durability or biosolubility is involved in fiber toxicity too. It consists in the ability of fiber to persist in the lung in spite of the physiological clearance mechanisms [16, 21]. Fiber durability is related to fiber biopersistence in the lung tissue. In particular, the biopersistence is the sum of physiological clearance processes (translocation, alveolar macrophage clearance, mucociliary escalator) and physicochemical processes that regard biosolubility (dissolution, leaching and breakage) [5].

Biosolubility of a fiber can be studied in vitro using models of simulated lung fluids and observing the loss of structural molecules or mass, expressed as dissolution constant (K_dis_).

These models have not been considered robust enough to be used for regulatory classification of fibers and are used as internal screening by the fiber industry in developing new fiber compositions [16, 22].

## Objective of the present study

In this work, acellular in vitro experiments were carried out. We studied the behaviour of four wools belonging to HTIW (RCF, two types of AES and PCW) in saline solution simulating physiological fluids. The study was carried out by measuring length and diameter of fibers pointing out morphological and chemical changes of fibers.

## Methods

### Choice of mineral wools

Four mineral wools belonging to HTIW were chosen. Three amorphous wools: RCF, a traditional wool used for very high temperature insulation applications and two AES wools that differ in the chemical composition. AES wools are an example of newly developed fibers, more biosoluble than traditional materials [2, 13]. The last sample was PCW, a polycrystalline wool. Their chemical composition is shown in Table 1.

**Table 1:** Chemical composition (% of weight) of the four mineral wools studied

	RCF	AES 1	AES2	PCW
SiO_2_	50–58	61–67	70–80	27
Al_2_O_3_	42–50	< 1	–	≥72
Fe_2_O_3_	–	< 0.6	–	–
MgO	–	2.5–6.5	> 18–27	–
CaO	–	27–33	–	–

The manufacturers provided the chemical composition ranges and not exact oxide content in fiber. In these cases, an average SiO_2_ content was considered in the calculation of dissolution constant.

The mineral wool samples have been prepared as described in the Draft-4 of European Chemicals Bureau method [23]. The bulk fibers are reduced in length using a crushing procedure in a 50 mm diameter die at 10 MPa. The material is mixed with spatula and repressed at 10 MPa and a small amount of sample (< 0.5 g) was dispersed in distillated water. A few milliliters are filtered through a 0.2 μm pore size, polycarbonate filter and prepared for examination using scanning electron microscope (SEM). The lengths and diameters were measured for 300 fibers.

### Stationary method

We used a static test according to the procedure defined in the Health and Safety Executive report [24]. About ten milligrams of the fibers, keeping the ratio of the surface area developed by the fibers to the volume of the solution roughly constant, were placed in polyethylene bottles containing 100 mL of Gamble’s solution Table 2.

**Table 2:** Composition of Gamble’s solution

Chemical	Weight (g/L)
NaCl	6.6
NaHCO_3_	2.7
CaCl_2_	0.002
Na_2_HPO_4_	0.358
Na_2_ SO_4_	0.08
MgCl_2_	0.211
Gly	0.118
Na_3_-citrate	0.153
Na_2_-tartrate	0.180
37% Formaldehyde solution	2 ml/L
Na-pyruvate	0.171
Na-lactate	0.175

The buffer solution was prepared in deionized water at two pH values (7.4 and 4.5). There are at least two pH environments in the lung: pH 7.4 simulates the near-neutral environment of the extracellular fluid and pH 4.5 mimics the acid environment of the phago-lysosomes of the macrophages [25]. Formaldehyde was added to prevent bacterial and algal growth. The experiment lasted four weeks. The initial solution had a starting pH of about 7.4; for pH 4.5 solution, HCl was added. Once a week pH was checked with a hand-held electronic pH meter. The bottles were kept at 37 ± 1 °C and shaken vigorously twice a day. The samples were left for 7, 14, 21 and 28 days. About 3 ml of each solution were filtered every seven days onto 0.8 μm polycarbonate membranes (25 mm diameter) so as to have enough fibers on the filter for SEM analysis without excessive fiber overlap. The membranes were dried in open tins, mounted on a stub and coated with gold under vacuum for SEM observation. Aliquots of each filtered solution were removed for measuring the concentrations of the elements extracted from each sample by inductively coupled plasma atomic emission spectrometry (ICP-AES).

### SEM analysis

Morphological and chemical changes of the fibers that accompanied the leaching and dissolution process were studied by SEM (LEO S440, LEO Electron Microscopy Ltd., Cambridge, UK) with energy-dispersive X-ray spectrometry (EDS, INCA ENERGY 400, Oxford Instruments, Abingdon, UK).

Dimensional analysis and chemical characterization were done for untreated and treated fibers in Gamble’s solution at two pH values. The lengths and the diameters were measured for 300 fibers of each sample by SEM.

### ICP-AES analysis

ICP-AES (Varian Vista MPX CCD Simultaneous ICP–AES, Varian Analytical Instruments, Mulgrave, Victoria, Australia) was used to determine the element concentrations released in solution [26].

Ultrapure deionized water from Milli-Q analytical reagent-grade water purification system (Millipore) and ultrapure HNO_3_ 1% were used. All the plastic lab ware employed, were metal free sterile polypropylene tubes, purchased from Perkin Elmer.

For the determination of the concentration of leached Si, Al, Ca and Mg from the fibers, a dilution was needed to avoid matrix effects [27–29]. Each filtered solution was diluted (1:10) with a 1% nitric acid solution, the most appropriate dilution found and analyzed by ICP. Gamble’s solution blanks were also measured, in the same analytical conditions as the samples.

The calibration solutions for ICP-AES quantitative measurements, were prepared from multi-element ICP Quality Control standards, purchased from Perkin Elmer: for Si (500 μg /ml) and Al, Mg, Ca (1000 μg/ml) (solutions in nitric acid matrix). All calibration solutions were matched by using element standards covering the concentration range of interest (0.005 mg/l to 100 mg/l). Standard solutions for daily five-point calibration were matrix-matched by preparation in Gamble’s solution to pH 4.5 and 7.4.

Quality control standards were analyzed every cycle of unknown samples and at the end of the analytical run. A rinsing time of 4 min with distilled water was used between the samples for each individual experiment. The element concentrations were converted into oxides.

The samples were analyzed in triplicate. An internal standard (yttrium 500 μg L-1 – wavelength 371.030 nm) was used to control nebulizer efficiency. The ICP-AES operating parameters, which were properly optimized to meet the appropriate detection limits, are given in Table 3.

**Table 3:** Analytical conditions for elemental analysis

ICP–AES instrumental parameters	
Incident plasma power (RF) (kW)	1*.*00
Plasma gas flow (L min^− 1^)	15*.*0
Auxiliary gas flow (L min^−1^)	1*.*50
Nebulizer pressure (kPa)	200
Replicate read time (s)	45
Instrument stabilization delay (s)	45
Sample uptake delay (s)	40
Pump rate (rpm)	20
Rinse time (s)	20
Replicates	3

The limit of quantification (LOQ, with a relative standard deviation equal to 5%) was reported for each element together with the reading and control wavelengths (Table 4).

**Table 4:** Wavelengths and LOQ

Element	Reading wavelength (nm)	Check wavelength (nm)	LOQ (μg/L)
Si	251.611	288.158	10.00
Al	396.153	396.143	8.00
Ca	315.887	317.933	5.00
Mg	279.553	279.077	4.00

### Dissolution rate

Dissolution rate (v), expressed in nm day^− 1^, is commonly quantified in terms of dissolution constant K_dis_, expressed in ng cm^−2^ h^− 1^. K_dis_ measures the ability of fibers to remain intact in simulated lung fluids and gives what is termed fiber durability [3, 30]. For vitreous fibers such as RCF and AES, the silicon is the principal component of network and K_dis_ can be estimated in terms of silicon mass lost through time. Its release in Gamble’s solution represents a valid parameter for the determination of fiber behaviour during dissolution process when it is a congruent dissolution (i.e. there is no selective leaching) [31]. The amount released in solution (%SiO_2_)_sol_ was obtained using eq. 1 of Thélohan and de Meringo [31]:

$$ {\left(\%{\mathrm{SiO}}_2\right)}_{\mathrm{sol}}=\left(\left[{\mathrm{SiO}}_2\right]\ \mathrm{V}\right)/\left(\mathrm{m}\ {\left(\%{\mathrm{SiO}}_2\right)}_{\mathrm{glass}}\right) $$

where [SiO_2_] = SiO_2_ concentration in the effluent solution (mg/L), V = volume of solution (L), m = mass of fibers tested (mg), (% SiO_2_) _glass_ = initial SiO_2_ content of fibers (weight percent). (% SiO_2_)_sol_ was measured weekly for each effluent solution by ICP analysis. The dissolution rate of the silica network, v, was calculated using eq. 7 by the same authors [31]:

$$ \mathrm{v}=\mathrm{d}/2\mathrm{t}\ \left(\ 1-\surd \left(1-{\left(\%{\mathrm{SiO}}_2\right)}_{\mathrm{sol}}\right)\right) $$

where d is the initial diameter of the fibers (untreated sample) and t is a determinate time of treatment of fibers in solution. The relationship between K_dis_ and v is:

$$ {\mathrm{K}}_{\mathrm{dis}}=\rho /0.24\mathrm{v} $$

where ρ = fiber density (g/cm^3^); 0.24 is the conversion factor from days to hours.

When elements such as alkali metals and alkali earths are present in the vitreous structure, they bind to oxygen atoms, breaking the silica network. These elements are referred to as network modifiers. Their presence increases the susceptibility of the fibers to dissolution. The network modifiers are weakly held in the glass structure and are leached faster than silicon. This leaching is referred to as incongruent dissolution.

The dissolution rate can be calculated similarly for leaching elements of glass oxides such as: Al_2_O_3_, CaO and MgO [32]. For PCW, not belonging to vitreous fibers, the network is formed mainly from aluminum and K_dis_ was calculated taking into account this element as well.

## Results

### SEM analysis: size parameters

Table 5 shows size parameters measured by SEM of untreated wools and after 28 days of treatment, for both pH values:

- D_LG_, is the length-weighted geometric mean diameter distribution of fibers, termed also accumulated length distribution, in which for each range of diameters the total length is specified and not the number as in usual fiber counting.
- d_G_, is the geometric mean diameter of fibers, defined as the diameter equivalent of the arithmetic mean of the logarithmic frequency distribution.
- l_G_, is the geometric mean length of fibers, defined as the length equivalent of the arithmetic mean of the logarithmic frequency distribution.
- σ is the geometric standard deviation, describes how spread out are a set of numbers whose preferred average is the geometric mean. It is a multiplicative factor, and thus dimensionless, rather than having the same dimension as the input values as the traditional standard deviation. The σ is used as a measure of log-normal dispersion similar to the way a geometric mean is used to measure central tendency.

**Table 5:** Size parameters of four samples analyzed. For each sample the parameters of untreated fibers (identified as std), treated fibers at pH 7.4 and 4.5 after 28 days of treatment are reported

	RCF			AES1			AES2			PCW		
	Std	pH 7.4	pH 4.5	Std	pH 7.4	pH 4.5	Std	pH 7.4	pH 4.5	Std	pH 7.4	pH 4.5
d < 3 μm (%)	69	67	72	55	–	39	66	64	56	20	24	20
d < 3 μm l > 5 μm (%)	68	65	71	53	–	39	62	62	55	20	24	20
d < 3 μm l > 20 μm (%)	44	37	49	27	–	28	19	24	28	11	12	9
D_LG_ (μm)	2.1	2.1	1.7	2.6	–	3.4	2.5	2.5	2.7	4.2	4.3	4.2
σ_LG_	2.2	2.1	2.7	1.9	–	2.0	1.9	2	2.1	1.4	1.5	1.5
d_G_ (μm)	2.0	1.5	1.8	2.6	–	3.9	2.2	2.1	2.5	4.0	3.9	3.9
σ_d_	2.3	3.2	2.3	2.0	–	2.6	2.0	2.0	2.1	4.0	1.5	1.4
l_G_ (μm)	31.4	31.1	33.3	23.3	–	28.5	17.2	22.2	26.7	25.6	25.7	22.6
σ_l_	2.3	2.6	2.3	2.2	–	2.5	2.1	2.3	2.3	1.6	1.7	1.6

Size parameters were not measured for AES1 at pH 7.4 because of complete dissolution of the fibers after 10/15 days of treatment

The geometric standard deviation, was calculated for each parameter (σ_LG_, σ_d_, σ_l_) using:

$$ \sigma =\exp \left(\sqrt{\frac{1}{N}{\sum}_{i=1}^N{\left(\ln \frac{xi}{\mu}\right)}^2}\right) $$

where N is the total number of fibers; x_i_ is a value of a data set (diameter/length) and μ is the geometric mean of the a data values.

### SEM/EDAX analysis: morphological and compositional changes

The untreated fibers were always smooth, with a regular surface, while the fibers corroded after the treatments had often a perturbed surface, which could take different shapes, and different surface composition.

**Fig. 1:**
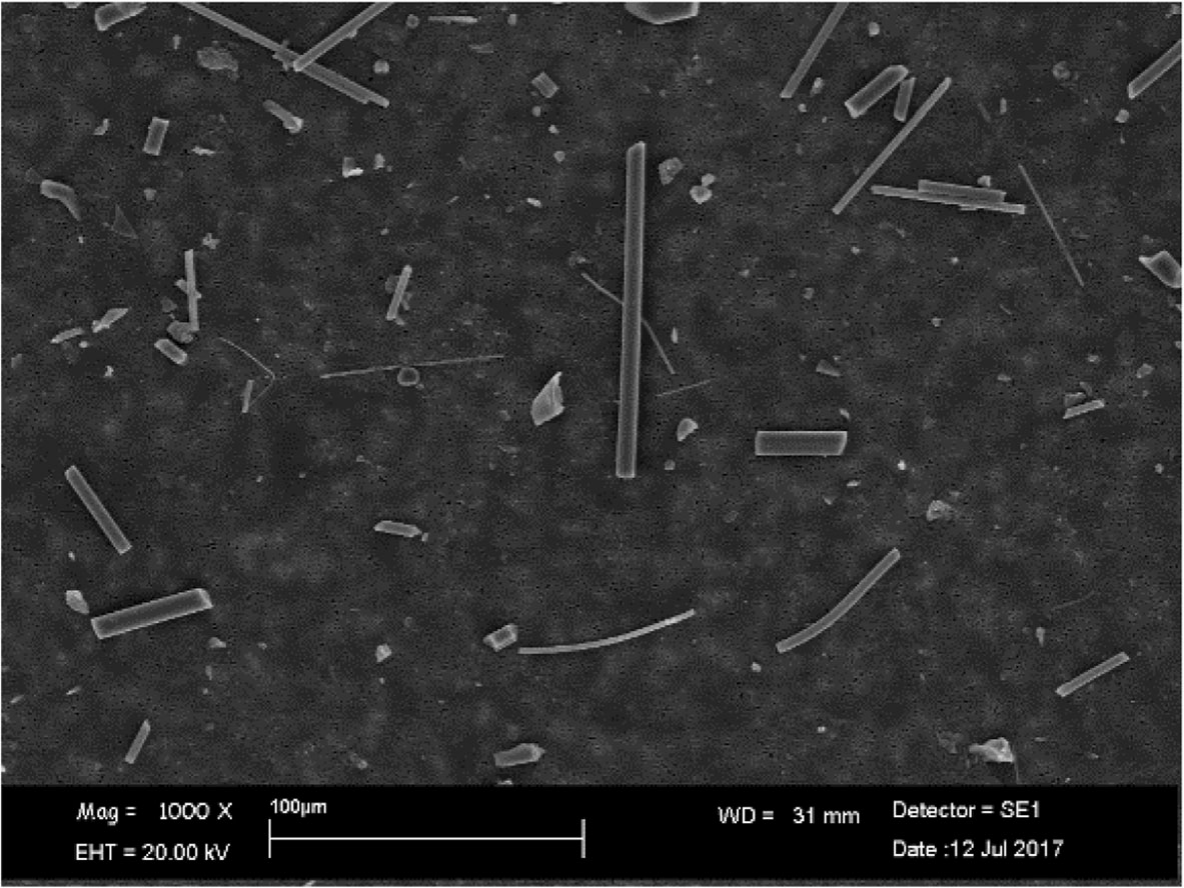
SEM image of RCF at pH 7.4 after 28 days of treatment

For RCF, the dissolution seemed to produce uncorroded fibers in both pH environments and they had the same chemical composition as the untreated fibers. The Fig. 1 shows RCF at pH 7.4 after 28 days of treatment. SEM image of untreated RCF, was not showed because there were no morphological changes with Fig. 1.

**Fig. 2:**
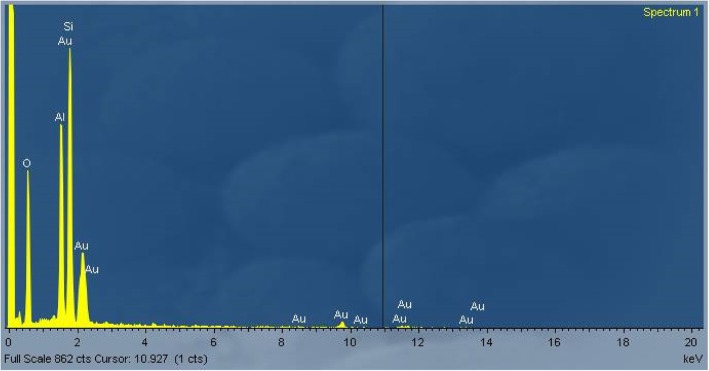
EDAX spectrum of RCF at pH 4.5 after 28 days of treatment

As an example, an EDAX spectrum of RCF after 28 days of treatment at pH 4.5 is reported in Fig. 2. The spectrum was the same for untreated RCF.

**Fig. 3:**
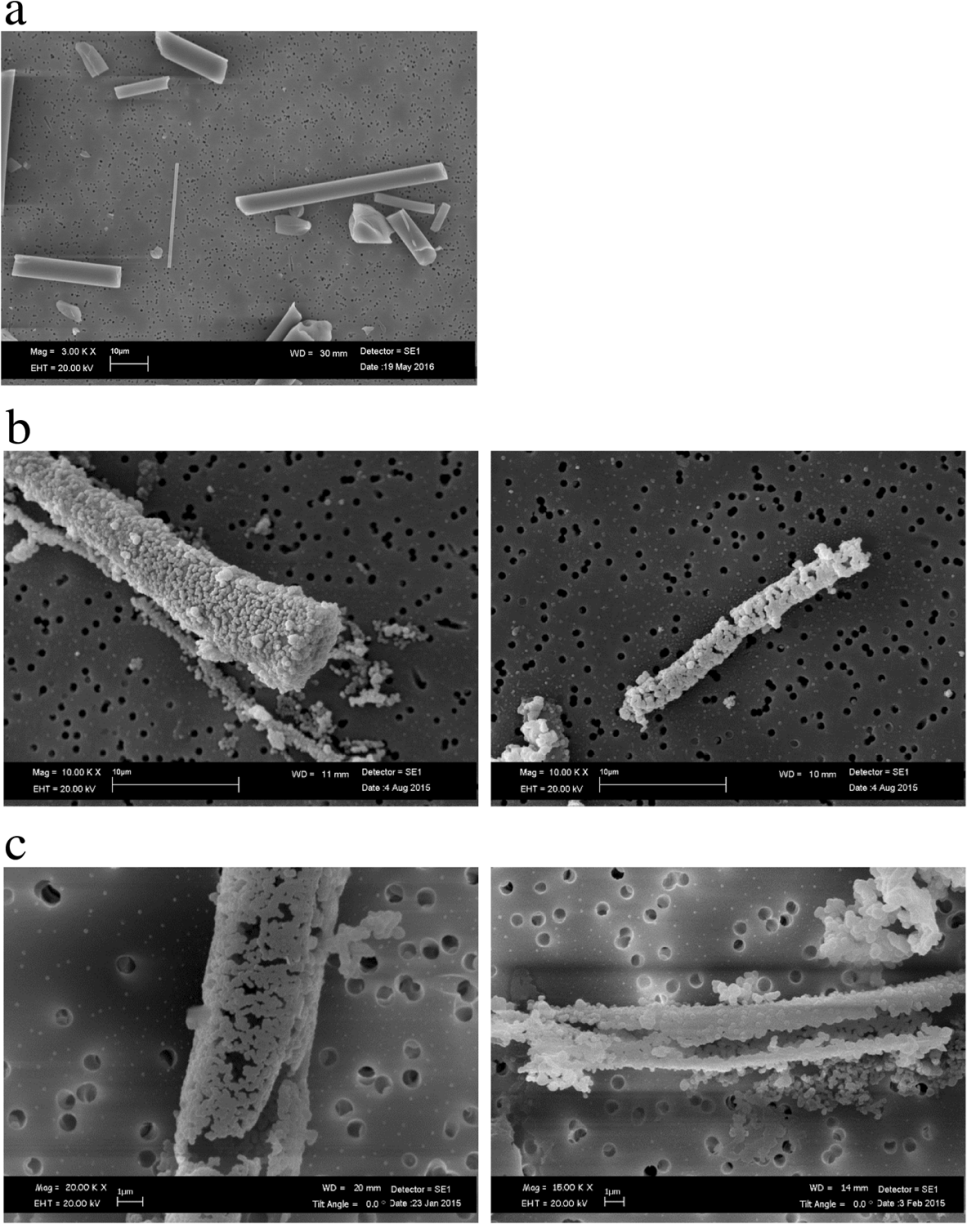
SEM images of AES1: (**a**) untreated fibers; (**b**) calcium phosphate precipitates on fiber surface layers at pH 7.4 after 28 days of treatment; (**c**) fiber imprint formed by calcium phosphate precipitates at pH 7.4 after 28 days of treatment

SEM analysis for AES1 showed a nearly complete dissolution (leaching process of calcium) of fibers after 28 days of treatment at pH 7.4 and formation of precipitates of calcium phosphate on the fiber surface of the (Fig. 3).

**Fig. 4:**
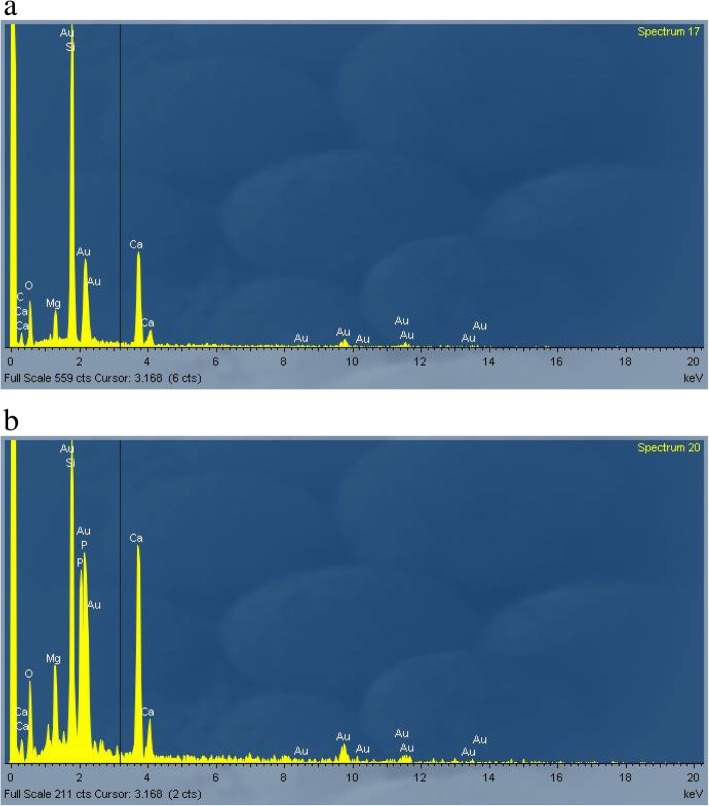
EDAX spectra of AES1: (**a**) untreated fibers; (**b**) fibers with calcium phosphate precipitates after 28 days of treatment at pH 7.4

Figure 4a shows EDAX spectrum of AES1 untreated fibers and the Fig. 4b EDAX spectrum of precipitates. Moreover, SEM observation of this wool allowed to detect that the complete dissolution of fibers happened between the first and the second week of treatment.

**Fig. 5:**
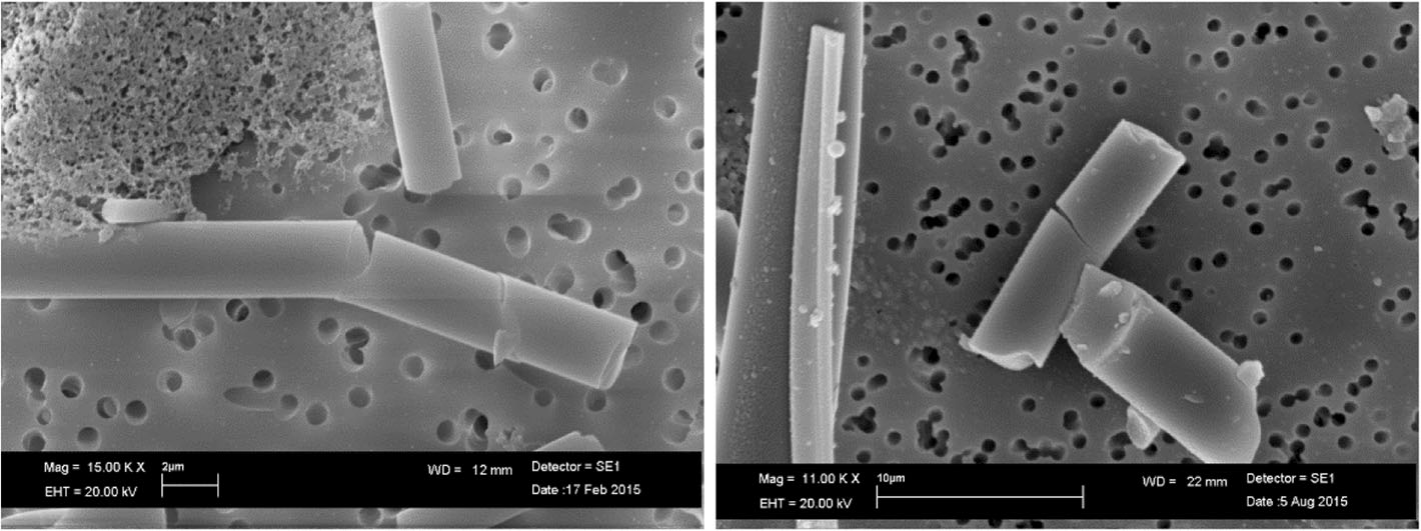
SEM image of AES1 transversal breakages at pH 4.5 after 28 days of treatment

At pH 4.5 AES1 fibers showed transversal breakages (Fig. 5) without involving chemical changes.

**Fig. 6:**
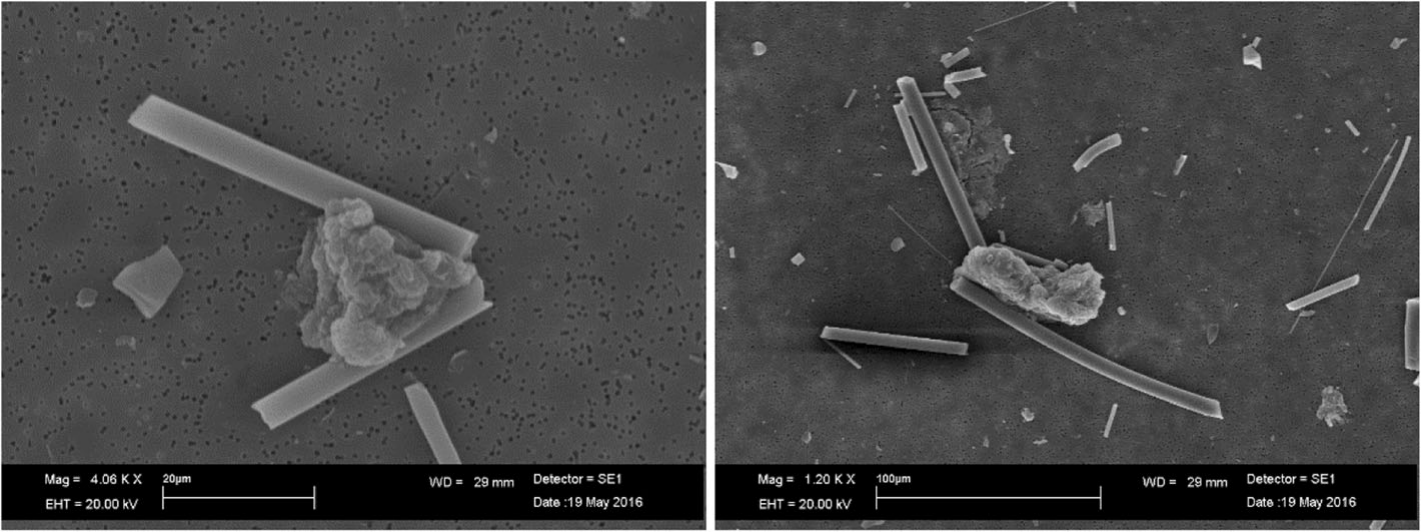
SEM image of AES2 at pH 7.4 after 28 days of treatment

For AES2 at pH 7.4 the dissolution process seemed to produce uncorroded fibers as showed in Fig. 6. SEM image of untreated AES2 fibers was not showed because there were no morphological changes and the image would been the same.

**Fig. 7:**
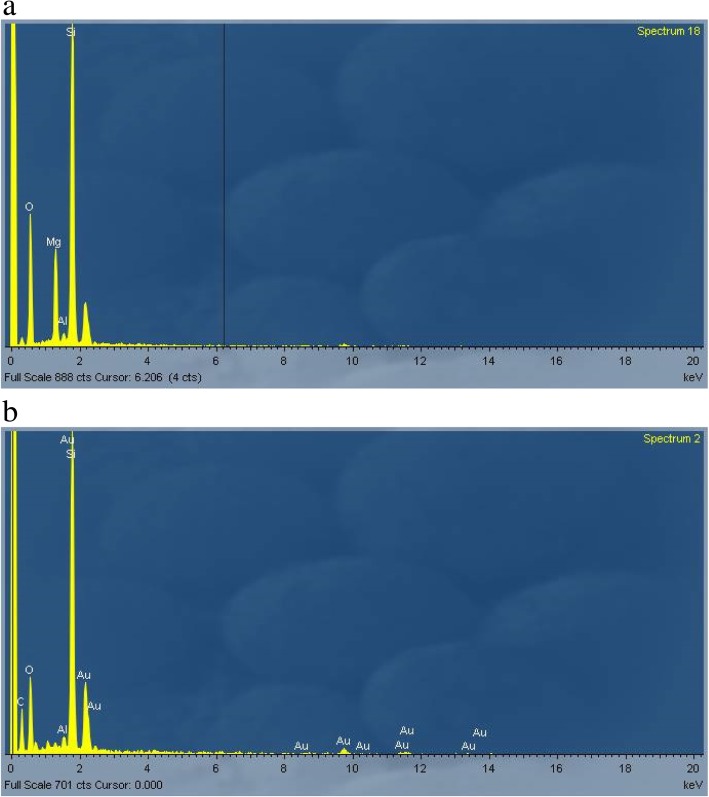
EDAX spectra of AES2: (**a**) untreated fibers; (**b**) some fibers after 28 days of treatment at pH 7.4 without magnesium peak

The chemical composition of untreated fibers (Fig. 7a) changed with a decrease or in some cases disappearance of magnesium peak as is shown in Fig. 7b.

**Fig. 8:**
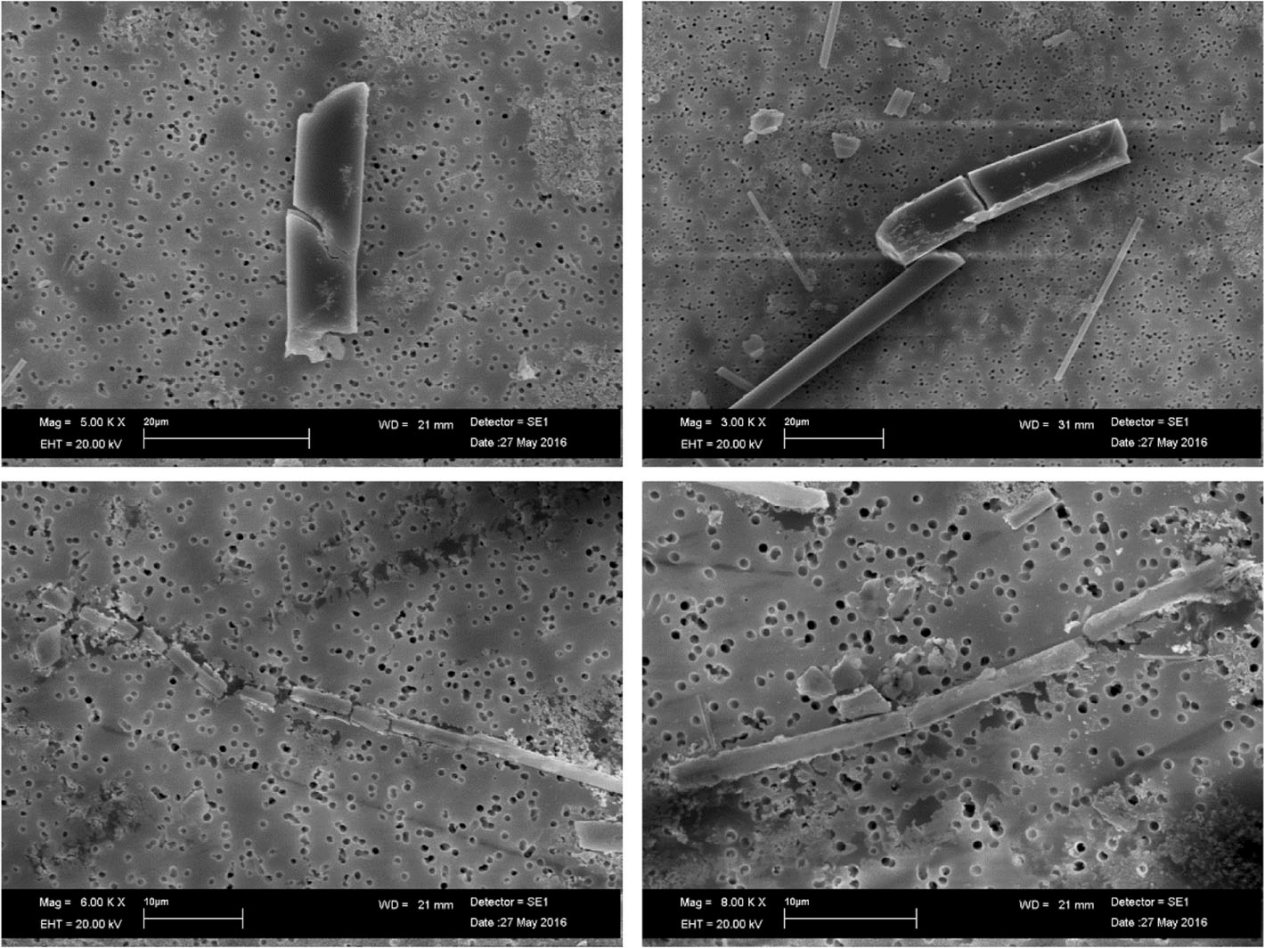
SEM image of AES2 transversal breakages at pH 4.5 after 28 days of treatment

In analogy to AES1, also AES2 at pH 4.5 showed several transversal breakages (Fig. 8) without involving chemical alterations.

**Fig. 9:**
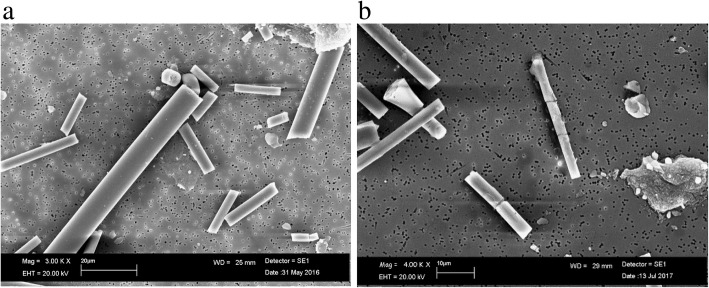
SEM image of PCW: (**a**) untreated fibers; (**b**) transversal breakages of fiber at pH 7.4 after 28 days of treatment

**Fig. 10:**
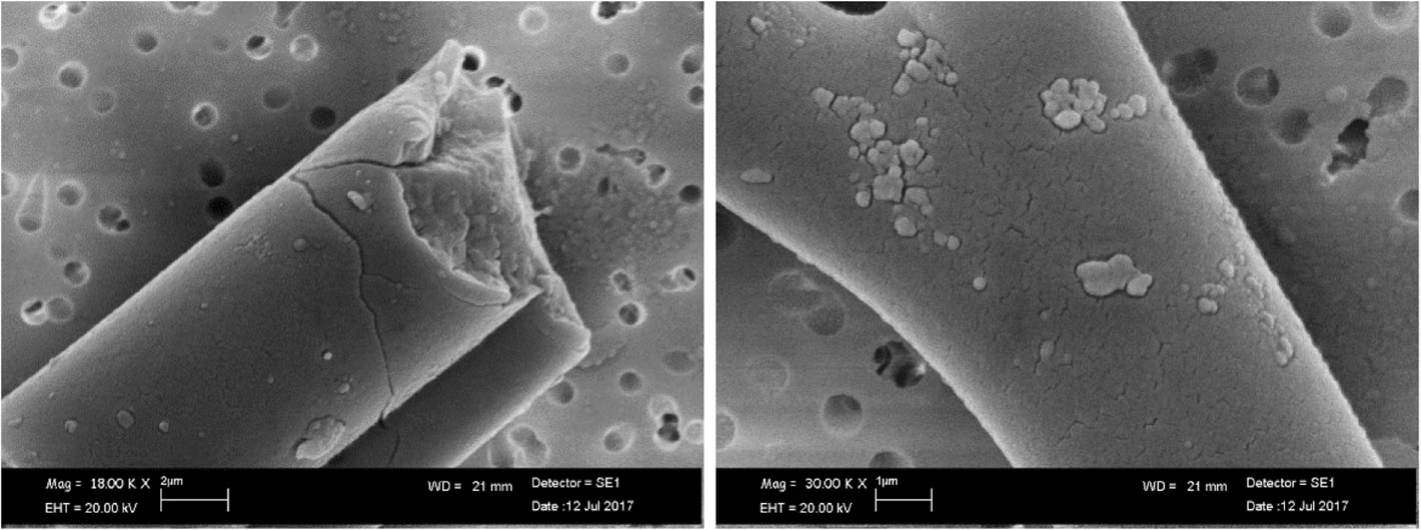
SEM image of PCW transversal breakages and surface weak corrosion at pH 4.5 after 28 days of treatment

PCW did not show morphological changes in physiological environment after 28 days of treatment compared to untreated fibers (Fig. 9). At acid environment, a weak corrosion on the fiber surface was observed (Fig. 10).

**Fig. 11:**
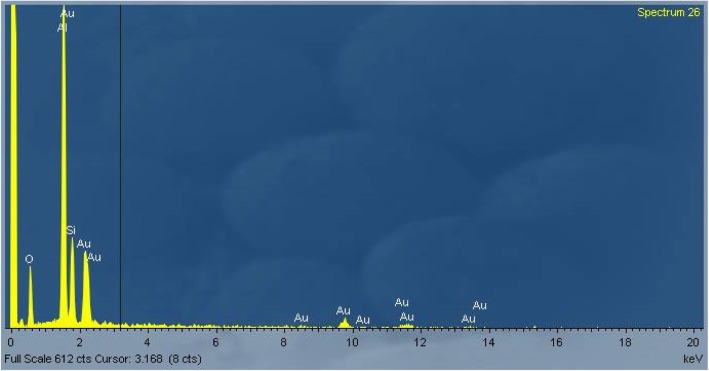
EDAX spectrum of PCW untreated fibers

The dissolution process led to transversal breakages of the fibers in both environments. Moreover, the fibers showed the same chemical composition of the untreated fibers (Fig. 11).

### ICP-AES analysis

Silicon, calcium, magnesium and aluminum concentrations in the effluent solution were measured by ICP-AES.

**Fig. 12:**
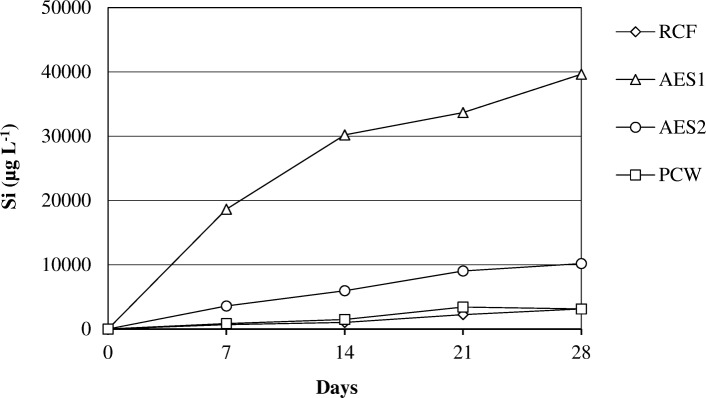
Dissolution of Si at pH 7.4

The amount of silicon release in the solution at pH 7.4 after 28 days of treatment was greater for AES1 than AES2 (Fig. 12).

**Fig. 13:**
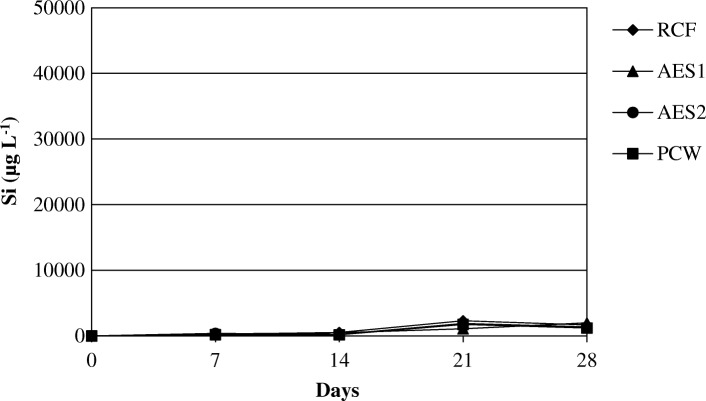
Dissolution of Si at pH 4.5

RCF and PCW, showed a similar behaviour in the same environment with a very low release of silicon in Gamble’s solution. At acid pH all samples showed the same trend relatively the silicon dissolution (Fig. 13).

**Fig. 14:**
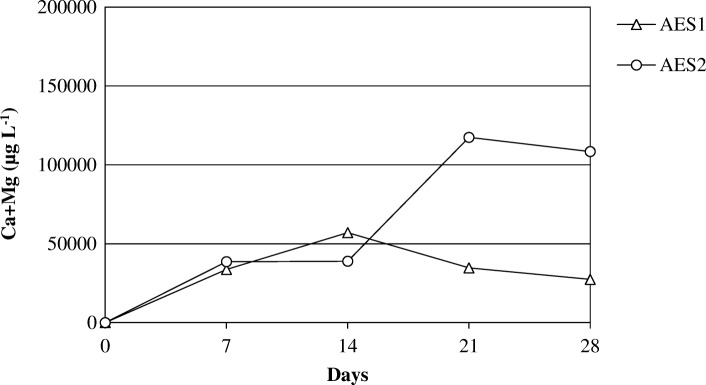
Dissolution of (Ca + Mg) at pH 7.4 for AES1 and AES2

**Fig. 15:**
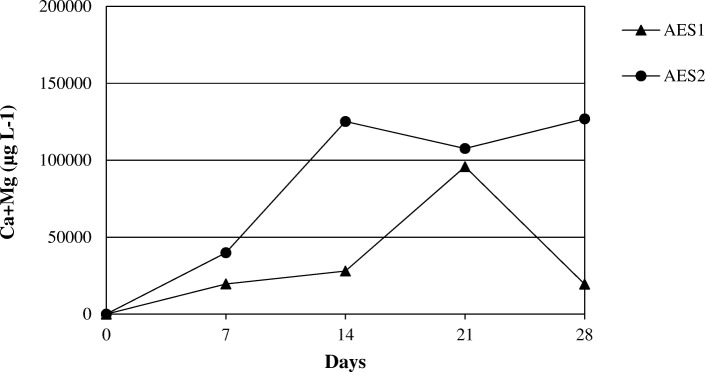
Dissolution of (Ca + Mg) at pH 4.5 for AES1 and AES2

**Fig. 16:**
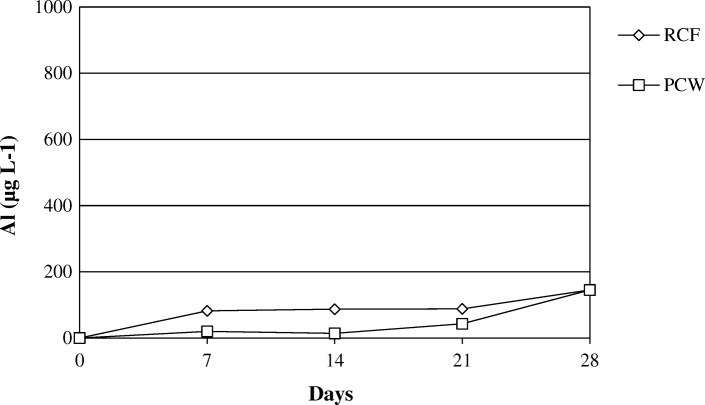
Dissolution of Al at pH 7.4 for RCF and PCW

**Fig. 17:**
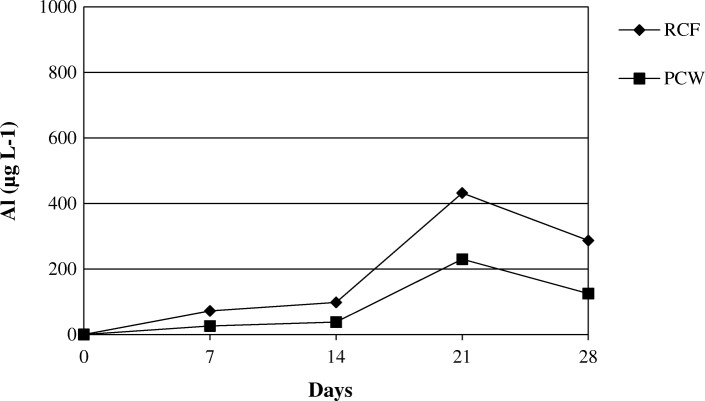
Dissolution of Al at pH 4.5 for RCF and PCW

The Figs. 14 and 15 show the release of calcium and magnesium in solution for AES1 and AES2 in physiological and acid environments respectively. The release of aluminum for RCF and PCW is shown in Figs. 16 and 17 in both environments.

### Dissolution rate

**Fig. 18:**
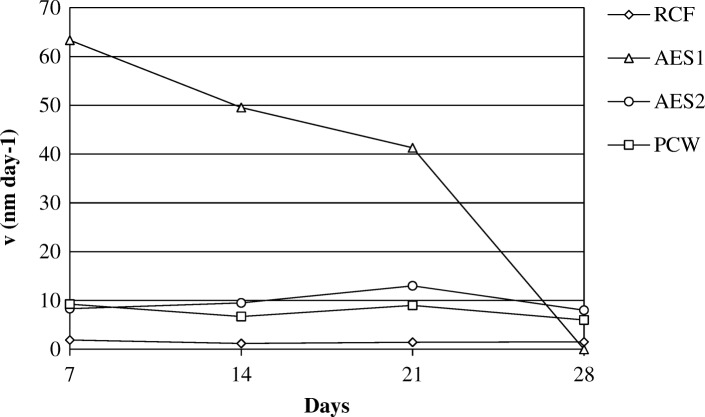
Dissolution rate at pH 7.4

**Fig. 19:**
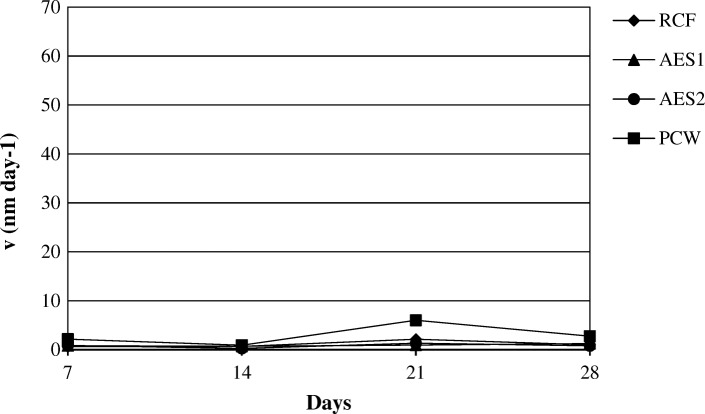
Dissolution rate at pH 4.5

The dissolution rates (v), are shown in Figs. 18 and 19 at two pH environments.

For AES1 and AES2, v was calculated based on the release of SiO_2_ dissolution while for RCF and PCW, v was calculated based on the release of SiO_2_ and Al_2_O_3_ dissolution. The Fig. 18 shows that AES1 is the sample with the highest dissolution rate in according to ICP analysis (Fig.12).

### Dissolution constant

K_dis_ values are expressed in ng cm^− 2^ h^− 1^, based on the dissolution rates [31]. It is assumed that K_dis_ is constant over time; a fiber can therefore be characterized by one dissolution rate constant calculated after 14–21 days of dissolution [21, 33, 34]. Table 6 shows the estimated K_dis_ over 14 days of treatment at two different pH values.

**Table 6:** K_dis_ (ng cm^− 2^ h^− 1^) calculated after 14 days of treatment in according to Thélohan and de Meringo [31]

	pH 7.4	pH 4.5
RCF	13	7
AES1	540	8
AES2	103	5
PCW	79	10

AES1 showed high solubility having high K_dis_ value at pH 7.4 after two weeks of treatment comparing to other samples. At acid pH, instead all samples showed a similar dissolution behaviour.

## Discussion

Four high temperature insulation wools were considered: AES1 and AES2 with different content of calcium and magnesium, RCF (aluminum silicate) and PCW (alumina/silica wools).

AES1 and AES2 are exonerated from the carcinogen classification in the EU under the terms of nota Q of CLP Regulation (2008) while RCF do not contain at least 18% alkaline earth oxides and fall under the classification of “category 2” carcinogen [8].

PCW are polycrystalline aluminum silicate fibers with a higher percentage of alumina compared to RCF. PCW are not vitreous and are not assessed under Directive 97/69/EC and CLP Regulation (2008) [8, 9]. IARC includes these wools as ceramic fibers group and considered them as “possible human carcinogens”, Group 2B [5].

The solubility tests, performed in this work, showed that AES1 (wools with high content of Ca) were more soluble than the other studied wools at pH 7.4, in accordance with our previous findings [3]. High value of K_dis_ (> 500 ng cm^− 2^ h^− 1^) suggests inhaled fibers are unlikely to be biologically relevant. Eastes and Hadley [35, 36] showed that no fiber with K_dis_ higher than 100 ng cm^− 2^ h^− 1^ produced fibrosis or tumors in animal inhalation studies. There is a tendency to consider 100 ng cm^− 2^ h^− 1^ as threshold to be free of respiratory disease. For AES1, showing high K_dis_ value, the long fibers would undergo complete dissolution and therefore would be removed more rapidly by clearance of alveolar macrophages [37].

The mechanism for clearance differs for short and long fibers. Short fibers are cleared by dissolution in the acidic environment of the phagolysosome within the macrophages and by macrophage removal. Long fibers are cleared by dissolution in the near neutral extracellular pH environment and breakage. For fibers that dissolve rapidly in acidic conditions, breakage may be facilitated by acidic conditions generated external to the cell by macrophages.

Both AES wools showed selective leaching of alkali/alkali earth elements at pH 7.4: a fast and extensive selective leaching of calcium for AES1 with complete dissolution of fiber already after 14 days of treatment and a moderate selective leaching of magnesium for AES2 in agreement to hypothesis of Searl [38]. This author discussed the role of chemical composition of glasses in dissolution process according to which the relative solubilities of glasses containing alkali and alkali earth oxides follow the solubility scale of the respective oxides: K_2_O > Na_2_O > Li_2_O > CaO > MgO.

The release of these elements in Gamble’s solution was greater for AES2 than AES1 in both environments after 28 days of treatment (Figs. 14 and 15). We would have expected the opposite, in view of complete fiber dissolution of AES1 in the physiological solution, but the formation of calcium phosphate precipitates on AES1 fibers subtracted calcium from the solution underestimating the concentration of this element.

All wools had a low K_dis_ at acid pH suggesting a low dissolution rate of short fibers (Fig. 13) [16].

Dissolution may proceed with or without evident morphological changes to the fibers. SEM analysis showed no evident size changes for RCF, AES2 and PCW in both pH values of solutions.

AES1 fibers showed an increase of D_LG,_ with d_G_ and l_G_, at acid pH after 28 days of treatment. The size distribution shifted to a larger mean diameter suggesting that through dissolution there is preferential loss of thin fibers. The amount of respirable fibers decreased from 53 to 39%.

Unlike the other wools, PCW being manufactured by sol-gel process, had a low content of respirable fibers. Sol-gel technology is designed to produce fibers of defined sizes (fiber mean diameter greater than 3 μm). Moreover this material had an initial low content of fibers with length greater than 20 μm. Long fibers are potentially relevant because there is considerable body of literature concerning the evidence that longer fibers (at least those longer than approximately 20 μm) are potentially more toxic in vivo than shorter fibers [14–16].

It was shown that for traditional wools (amorphous materials) with a high aluminum content had low solubility at pH 7.4 [39, 40]. In contrast, in an acid environment, fibers with an increased content of Al_2_O_3_ and decreased content of SiO_2_ had a high dissolution rate [41]. The dissolution behaviour of PCW (crystalline fibers with high aluminum and low silicon content) did not follow what has just been described. PCW showed a low congruent dissolution at pH 7.4 (K_dis_ = 79 ng cm^− 2^ h^− 1^) and not dissolving at pH 4.5 (K_dis_ = 10 ng cm^− 2^ h^− 1^). For RCF, as expected, are not dissolved at both pH environments.

The K_dis_ value of PCW measured at pH 7.4 was slightly higher than value of RCF. This difference is due to the slight changes of both chemical and size parameters [31].

At acid pH, both the amorphous (AES) and crystalline (PCW) fibers broke transversely (across the fiber), resulting in fibers which are of the same diameter as the original fiber but shorter (thus more easily removed by macrophages). The propensity of fibers to break transversely is important in determining their pesistence in the lung because shorter fibers can be more readily cleared by macrophages [12]. No fragmentation of RCF was observed.

Unlike the AES wools, RCF and PCW showed virtually no change in composition with time under either pH condition.

The static test used in this work has more limitations than flow-through measurements, such as restricted volume of the solution that can became supersaturated, or alteration of chemical composition and pH of solution. It was discussed the criticality of using small volume of solution in static test and was observed that the fibers tested in static and flow-through system showed a similar degree of dissolution (that is, between very rapidly dissolving fibers, fairly rapidly dissolving fibers and fibers that dissolve relatively slowly) [42].

Some studies showed that both methods had a similar degree of discrimination between fiber types and similar relative dissolution rate [34, 43, 44]. Moreover the dissolution rates for different fibers were in the same relative order as those obtained by the flow-through method [45].

Our estimated K_dis_ values are in agreement with the results found in literature. K_dis_ for AES1 is greatly underestimated because of the deposits on the fiber inhibiting its successive dissolution. Higher values (about 900 ng cm^− 2^ h^− 1^) were found by Hesterbeg et al. [46]. If we extrapolate K_dis_ data of Fig. 18 until few days of dissolution treatment (corresponding to about the time in which the deposits are probably absent), the value rises to over 800 ng cm^− 2^ h^− 1^. Moreover, using the rat inhalation model, Hesterberg et al. [46] showed that fibers with the same composition of AES1 underwent a decrease of calcium in the lung environment, as observed in this work.

## Conclusion

Among the four types of HTIW wools studied, AES wools were the most soluble at physiological pH. AES1 with high calcium content showed greater solubility than AES2 with high magnesium content in according to the solubility scale of their respective oxides. These wools showed a high degree of leaching, that is elements such as calcium and magnesium were removed more rapidly than other (incongruent dissolution). As a result of leaching, these fibers underwent compositional change and transverse fragmentation.

The dissolution process for PCW led to breakage transverse in both pH environments (low congruent dissolution). Transverse breakage of long fibers in the lung would increase the probability of clearance by macrophages [47].

For RCF, the treatment produced uncorroded fibers in both pH environments without chemical and fiber fragmentation (no dissolution).

The estimated K_dis_ at physiological pH follow the sequence: AES1 > AES2 > PCW > RCF. All wools had a low K_dis_ at acid pH suggesting a low dissolution rate of short fibers.

In the gas exchange region of the lung, simulated from a solution at physiological pH, the main mechanism for clearance of fibers is engulfment by alveolar macrophages. If the fibers are too long, the process is frustrated. Generally, fibers with length greater than 20 μm are not cleared from the lungs by alveolar macrophages but can cleared through dissolution and transversal fragmentation. For AES1 that showed high values of K_dis_, the macrophage-mediated clearance of long fibers (> 20 μm) was negligible and the principal clearance mechanism were dissolution and transverse breakage.

In addition to simple dissolution, also leaching and transverse fragmentation play an important role in the biopersistence mechanisms and pathogenicity of fibers.

Dissolution rate constant K_dis_ calculation is undoubtedly useful as a preliminary toxicological screening of fibers, especially for developing fibers.

Although the static test used in this work is far from reproducing what occurs in vivo, it is easy to carry out, quite fast and low cost method. This test can be regarded categorizing tool of different fibers for distinguishing between fibers that dissolve readily in the lung and relatively insoluble fibers but it is not able to provide a reliably ranking of solubility. Generally, the fibers that dissolve readily are materials with lower biodurability and are associated with a lower pathogenicity. The dissolution tests can provide information on risk assessment in order to prevent the exposure effects in the workplaces.

Further in vitro and in vivo studies are necessary to clarify the possible toxicity of these materials for their safe use.

## Abbreviations

AES: Alkaline earth silicate wools
EDS: Energy-dispersive X-ray spectrometry
ICP-AES: Inductively coupled plasma atomic emission spectrometry
K_dis_: Dissolution constant
PCW: Polycrystalline wools
RCF: Refractory ceramic fibers
SEM: Scanning electron microscopy
